# The Interaction Between Sleep and Metabolism in Alzheimer’s Disease: Cause or Consequence of Disease?

**DOI:** 10.3389/fnagi.2019.00258

**Published:** 2019-09-20

**Authors:** Caitlin M. Carroll, Shannon L. Macauley

**Affiliations:** Section of Gerontology and Geriatric Medicine, Department of Internal Medicine, Wake Forest School of Medicine, Winston-Salem, NC, United States

**Keywords:** Alzheimer’s disease, type-2-diabetes, sleep, glucose, insulin, metabolism

## Abstract

Alzheimer’s disease (AD) is the most common form of dementia and affects over 45 million people worldwide. Both type-2-diabetes (T2D), a metabolic condition associated with aging, and disrupted sleep are implicated in the pathogenesis of AD, but how sleep and metabolism interact to affect AD progression remains unclear. In the healthy brain, sleep/wake cycles are a well-coordinated interaction between metabolic and neuronal activity, but when disrupted, are associated with a myriad of health-related issues, including metabolic syndrome, cardiovascular disease, T2D, and AD. Therefore, this review will explore our current understanding of the relationship between metabolism, sleep, and AD-related pathology to identify the causes and consequences of disease progression in AD. Moreover, sleep disturbances and metabolic dysfunction could serve as potential therapeutic targets to mitigate the increased risk of AD in individuals with T2D or offer a novel approach for treating AD.

## Introduction

Alzheimer’s disease (AD) is severe neurodegenerative disorder characterized by the accumulation of extracellular amyloid plaques and intracellular neurofibrillary tau tangles (NFT), with detectable pathology occurring 15–20 years prior to the onset of clinical symptoms [as reviewed in Holtzman et al. (2011)]. Deposition of amyloid-beta (Aβ) into amyloid plaques is one of the initial changes observed in presymptomatic AD, with tau hyperphosphorylation, aggregation, and NFT formation to follow in spatially distinct regions (Bateman et al., 2012). Although changes in cerebral amyloid burden occurs early and is necessary for an AD diagnosis, the development of tau pathology, both temporally and spatially, more closely aligns with neurodegeneration and clinical symptoms. In general, disease development is multifaceted and influenced by a variety of genetic, environmental, and lifestyle components. Brain hypometabolism, hyperexcitability, oxidative stress, mitochondrial dysfunction, brain insulin resistance, and inflammation all contribute to AD pathophysiology, yet it is unclear whether they are a cause or consequence of Aβ and tau pathology (Musiek and Holtzman, 2015). Type-2-diabetes (T2D), a metabolic disease also associated with aging, is characterized by hyperglycemia, hyperinsulinemia, insulin resistance, and beta cell dysfunction. Sleep, on the other hand, is a complex homeostatic mechanism tied to altered consciousness and decreased responsiveness to sensory stimuli that relies heavily on the tight coupling between cerebral metabolism and neuronal activity. Both T2D and sleep disorders are implicated in the pathogenesis of AD, but the exact role sleep and metabolism play remains unclear (Ott et al., 1999; Stanley et al., 2016). In this review, we summarize the current understanding of the interplay between metabolism, sleep, and AD pathophysiology with an emphasis on disrupted sleep as a modifiable lifestyle characteristic contributing to increased risk of both AD and T2D.

## Glucose Metabolism and Neuronal Activity in AD

The relationship between metabolic activity and neuronal activity is central to both healthy brain function as well as the pathogenesis of AD. First, neuroimaging studies confirmed that prior to the hypometabolism typically described in preclinical stages of AD, brain regions most vulnerable to Aβ and tau accumulation are uniquely reliant on glucose for normal brain function and display increased glucose consumption at a young age (Edison et al., 2007; Mosconi et al., 2008; Vaishanavi et al., 2010; Vlassenko et al., 2010; Oh et al., 2016; Hanseeuw et al., 2017; Adams et al., 2018). Aerobic glycolysis is an emerging biomarker of network vulnerability in AD where brain regions prone to Aβ deposition utilize glucose beyond what is necessary for energy production, even though sufficient concentrations of oxygen exist for oxidative phosphorylation and ATP generation (Vlassenko et al., 2010, 2018; Yao et al., 2011; Vlassenko and Raichle, 2015; Harris et al., 2016). Biosynthesis, synaptic plasticity, synaptic remodeling, memory formation, and other key processes utilize glucose in this way and are essential for healthy brain function. In AD, brain regions predisposed to Aβ deposition markedly overlap with the brain’s default mode network, a network characterized by highly variant resting-state blood oxygen level dependent fMRI signals, high resting-state functional connectivity, high aerobic glycolysis, and high neuronal activity (Jagust and Mormino, 2011). In amyloid-positive, cognitively normal adults, elevated default mode network connectivity patterns are associated with low tau burden, while decreased connectivity is associated with increased tau burden, introducing a pattern of hyper- and hypoconnectivity along the preclinical-AD trajectory (Schultz et al., 2017). Recent studies demonstrate that individuals with high amyloid burden and lower aerobic glycolysis also have higher tau burden, another core pathological feature of AD (Vlassenko et al., 2018). Therefore, an interesting relationship emerges that links brain regions that are both metabolically and neuronally active with regions susceptible to AD pathology. This places regional glucose metabolism in the aging brain at the forefront of AD pathogenesis.

In a metabolically healthy brain, neuronal activity evokes a localized vascular response to increase cerebral blood flow and deliver enough oxygen and glucose to sustain periods of increased activity. Therefore, regional glucose consumption and glucose concentration in the brain’s interstitial fluid (ISF) are closely tied to changes in neuronal activity. However, it is still unclear how alterations in glucose availability and glycemic variability can modify the relationship between neuronal and metabolic activity to influence AD-related pathology and dementia. Elevated blood glucose levels, or hyperglycemia, are associated with an increased prevalence of dementia and AD, faster progression from mild cognitive impairment (MCI) to AD, and increased rates of Aβ accumulation (Whitmer et al., 2009; Aung et al., 2012; Crane et al., 2013; Lin and Sheu, 2013; Yaffe et al., 2013; Morris et al., 2014; Gomez et al., 2018). Similarly, repeated episodes of hypoglycemia that typically accompany treatment of T2D are also associated with increased risk of dementia and dementia of an Alzheimer’s type. In preclinical models, inducing peripheral hyperglycemia in rodents increases hippocampal ISF glucose, Aβ, and lactate levels. This effect is exacerbated in mice with significant Aβ pathology, demonstrating fluctuations in peripheral metabolism not only alter cerebral metabolism and Aβ release but amyloid plaques impact the brain’s response to glycemic changes (Macauley et al., 2015). Moreover, fluctuations in ISF lactate accompany hyperglycemia and represent changes in neuronal activity (Pellerin and Magistretti, 1994). Evidence from the astrocyte neuron lactate shuttle (ANLS) demonstrates glutamate release from neurons stimulates glycolysis within astrocytes, causing astrocytes to shuttle the glycolytic end-product, lactate, to neurons as fuel for ATP generation. Thus, preferential utilization of lactate over glucose occurs during periods of heightened neuronal activity and relative changes in the pool of ISF lactate represents changes in excitatory neurotransmission. Indeed, previous studies show that Aβ is released in an activity-dependent manner via changes in synaptic vesicle exocytosis (Cirrito et al., 2005) and increased neuronal activity is coupled to ISF lactate and Aβ levels. Moreover, regional differences in Aβ levels and subsequent plaque formation mirrors regional patterns of neuronal activity, where hyperexcitability is associated with increased plaque aggregation (Busche et al., 2008; Bero et al., 2011). Although tau is a cytoplasmic protein and pathological tau accumulates intraneuronally, tau is found in the ISF and cerebrospinal fluid (CSF) demonstrating it can be released from neurons. Historically, extracellular tau was thought to be released from dead or dying cells, but recent studies demonstrate, similar to Aβ, tau is released during neuronal activity under normal conditions (Pooler et al., 2013; Yamada et al., 2014), and accumulates in periods of hyperactivity (Huijbers et al., 2019). Extracellular tau monomers and aggregates can be taken up by neighboring neurons where tau pathology can propagate and spread through network connectivity (Clavaguera et al., 2009; Lehmann et al., 2013; Wu et al., 2016; Franzmeier et al., 2019). Therefore, mechanisms that alter neuronal excitability also modulate the levels of Aβ and tau in the extracellular space and over time, can promote or attenuate Aβ and tau aggregation. This has been shown in clinical epilepsy populations (Mackenzie and Miller, 1994; Tai et al., 2016) and *APOE4* targeted replacement mice, a genetic knock-in model of AD (Klein et al., 2014; Nuriel et al., 2017), where network hyperexcitability leads to increased accumulation of AD pathology. Taken together, these data support a reciprocal relationship between metabolic activity, neuronal activity, and AD-related proteins.

Individuals with T2D have a 2–4-fold increased risk of developing AD (Ott et al., 1999). Although the relationship between T2D and AD is complex, alterations in glucose metabolism and glucose homeostasis are central to both diseases. As a disease associated with aging, T2D is characterized by hyperglycemia, hyperinsulinemia, and insulin resistance (Ott et al., 1999). While disrupted glucose regulation is correlated with cerebral hyperexcitability and accumulation of pathology, disrupted insulin signaling and insulin resistance also have strong associations with MCI, dementia, and AD (Craft, 2007; Talbot et al., 2012). Further, there is evidence that insulin resistance may be an early biomarker of AD risk in cognitively normal individuals, as greater insulin resistance is correlated with decreased cerebral glucose uptake and poor memory performance (Baker et al., 2011). Peripheral hyperinsulinemia leads to increased circulating Aβ and CSF Aβ42 in cognitively normal adults (Watson et al., 2003; Fishel et al., 2005) and increased hyperphosphorylated tau protein expression in mice (Freude et al., 2005). Further, in rodent models, hyperinsulinemic-euglycemic clamps raised serum insulin levels and modestly impacted ISF Aβ levels without detectable changes in insulin levels or signaling pathways in the brain (Stanley et al., 2016). Further, chronic hyperinsulinemia is thought to lead to lower insulin levels in the brain, which could explain why treating with insulin has been shown to cause memory improvements (Craft et al., 1996; Kern et al., 2001; Rerger et al., 2008). These studies suggest that both abnormal peripheral glucose metabolism and insulin resistance, akin to that seen in T2D, can accelerate AD pathogenesis through alterations in both neuronal and metabolic activity.

## Sleep Architecture and Metabolic Regulation

Sleep is a conserved behavior that occurs in two distinct stages – non-rapid eye movement (NREM) and rapid eye movement (REM). NREM sleep is characterized by large, slow waves that propagate across the entire cortex, producing synchronized periods of neuronal activation and silence (Steriade et al., 1993). It is hypothesized that this slow wave activity (SWA) of NREM sleep is restorative in nature; a price paid for neuronal activity and synaptic strengthening that occurs during waking, and therefore representative of the homeostatic need for sleep (Vyazovskiy et al., 2009). SWA peaks early in the night and increases following periods of extended wakefulness and decreases across sleep periods as well as following daytime naps (Werth et al., 1996; Achermann and Borbley, 2003). This is correlated to the patterns of neuronal activity across the sleep/wake cycle where firing rates are high during waking and lower across sleep, therefore indicating a relationship between synaptic activity and sleep (Vyazovskiy et al., 2009). Further, periods of heightened neuronal activity and synaptic strengthening cause local increases in SWA, while stimulus deprivation or pharmacological depotentiation of synapses causes a reduction in SWA, further demonstrating SWA as a marker for homeostatic sleep need (Huber et al., 2006, 2007; Carroll et al., 2019).

Glucose and lactate also show consistent patterns across the sleep/wake cycle closely related to neuronal activity. Glucose concentrations in the brain increase during both NREM sleep, which is thought to aid in restoration and replenishment of glucose stores, and waking, indicative of increased metabolic functioning. While glucose cannot accurately measure sleep propensity because of these increases across both states, it is useful as a biomarker for cerebral metabolism (Dash et al., 2012b). Lactate concentrations in the brain increase with heightened neuronal activity during wakefulness and decrease across the night in correlation with both SWA and glutamate concentration, suggesting a shift toward astrocytic glycolysis and lactate utilization in periods of increased neuronal activity. Because of this tight correlation with neuronal activity, lactate is hypothesized to be an accurate biomarker for homeostatic sleep (Dash et al., 2012b; Naylor et al., 2012).

In the periphery, blood glucose is homeostatically regulated to maintain euglycemia and prevent extreme fluctuations. Glucose tolerance reflects both hepatic gluconeogenesis and glucose utilization and it is closely related to insulin release from pancreatic beta cells, insulin sensitivity of peripheral tissues, or the ability of insulin to inhibit glucose production and increase glucose utilization. With decreased insulin sensitivity or insulin resistance, the body is unable to effectively combat hyperglycemia, allowing for consistent states of high blood glucose (Knutsson et al., 2007). Blood glucose is also regulated by a circadian rhythm where plasma glucose levels are higher in the evening than morning and glucose tolerance and insulin sensitivity are lowest at night (Van Cauter et al., 1997). As the brain utilizes the majority of glucose in the body, peripheral and cerebral glucose levels are tightly correlated and shifts in peripheral glucose levels tend to model shifts in patterns of neuronal activity across the sleep/wake cycle. As such, glucose utilization is increased during wakefulness to accommodate for high levels of neuronal activity (Nofzinger et al., 2002). ISF glucose also increases across NREM sleep, likely reflecting both a decrease in glucose utilization as well as a decrease in insulin sensitivity (Spiegel et al., 2009; Dash et al., 2012a). Further, increasing concentrations of glucose can promote slow wave sleep through increased activation of the sleep-promoting neurons in the ventrolateral preoptic nucleus (VLPO) (Varin et al., 2015), as well as inhibiting hypothalamic orexin neurons that typically promote arousal (Yamanaka et al., 2003). As described earlier, glucose dysregulation and insulin resistance are key components connecting AD and T2D, so dysregulation of normal sleep/wake rhythms could be mediating this relationship between the two diseases.

## AD and Sleep

Disrupted sleep is commonly observed in individuals with symptomatic AD (Peter-Derex et al., 2015) and can be detected in the preclinical stage (Ju et al., 2013; Hahn et al., 2014; Lucey et al., 2019). Moreover, sleep fragmentation, poor sleep quality, and excessive daytime sleepiness have been shown to increase risk of cognitive impairment and eventual dementia in clinical populations (Blackwell et al., 2013; Lim et al., 2013a; Spira et al., 2015, 2018). One of the most common causes of sleep disturbances in older adults is sleep disordered breathing (SDB), largely due to the high prevalence of obstructive sleep apnea (OSA) in the elderly (Andrade et al., 2018). In OSA, the airway is blocked during sleep, causing difficulty breathing, reduced blood oxygen saturation (e.g., hypoxemia), recurrent arousals, and sleep fragmentation (Yaffe et al., 2011; Osorio et al., 2015; Sharma et al., 2018; Ju et al., 2019). Cross-sectional and prospective studies in humans both demonstrate individuals with OSA are at increased risk for cognitive impairment, dementia, and changes in AD-related biomarkers (Yaffe et al., 2011; Ju et al., 2019). Conversely, treatment for OSA with continuous positive airway pressure (CPAP) reduces sleep fragmentation, hypoxia, and normalizes CSF Aβ/tau levels. In rodent models, acute sleep disruptions, as modeled in mice via chronic intermittent hypoxia, causes impaired spatial learning and memory (Ward et al., 2009), as well as increased Aβ and tau pathology (Gao et al., 2013; Shiota et al., 2013). This established a bidirectional relationship between sleep, cognition, and AD pathogenesis, which is further supported by the general characteristics of sleep and resulting fluctuations in Aβ and tau. As Aβ and tau are released in an activity dependent manner, the levels of Aβ and tau within the ISF of the brain parallel the diurnal rhythm of neuronal activity across the sleep/wake cycle. ISF Aβ and tau are closely correlated with wakefulness, with increases during sleep deprivation in both mice and humans (Kang et al., 2009; Lucey et al., 2017; Holth et al., 2019). Further, sleep has also been associated with the clearance of Aβ/tau from the ISF into the CSF and blood (Xie et al., 2013). Any situation that disrupts sleep, therefore, will alter Aβ and tau production and clearance. Decreased sleep spindle density, for example, has been connected to early neuronal dysfunction, particularly as it relates to tau release (Kam et al., 2019). Similarly, sleep deprivation increases Aβ and tau levels acutely and can lead to increased plaques, tangles, and cognitive decline in chronic conditions (Kang et al., 2009; Spira et al., 2013; Ju et al., 2017; Lucey et al., 2017; Zhu et al., 2018; Holth et al., 2019). Restoring sleep, however, has been shown to reduce ISF Aβ and tangle density (Kang et al., 2009; Lim et al., 2013b; Tabuchi et al., 2015). While the exact mechanism is unknown, increased neuronal activity could be playing a role in increasing AD pathology, as chronic sleep deprivation has been shown to increase intrinsic neuronal activity (Tabuchi et al., 2015). Because of the strong diurnal pattern of neuronal activity across the sleep/wake cycle, lactate, a biomarker for neuronal activity, is also used as a biomarker for sleep (Naylor et al., 2012). The diurnal oscillation of lactate, therefore, mirrors that of neuronal activity and Aβ, providing evidence that neuronal activity, mediated by the sleep/wake cycle, is responsible for driving AD pathology accumulation, particularly during sleep deprivation (Roh et al., 2012). While it is unknown what causes the initial disruption in sleep, degeneration of certain sleep-related nuclei, including the VLPO and intermediate nucleus, has been associated with sleep loss in AD (Lim et al., 2014). Moreover, recent work has shown individuals with MCI and AD have elevated levels of orexin in the CSF, which could be contributing to the overall increase in arousal seen in these individuals (Gabelle et al., 2017). This initial disruption could activate a cascade of accumulating sleep debt and pathology accumulation and explains the epidemiological evidence connecting sleep disturbances and AD.

## Sleep Disruption and Metabolic Disturbances

Poor sleep quality and quantity is also a common feature of individuals with T2D (Spiegel et al., 1985; Yaggi et al., 2006), and can increase the risk of developing T2D by 2–3-fold (Kawakami et al., 2004). Hypoglycemia alone has been shown to activate orexin neurons in the hypothalamus, causing increased arousal in both mice and men (Tkacs et al., 2007). This effect is ablated in individuals with T2D, where hypoglycemia episodes failed to increase arousal, leading to poorer glycemic control and suggesting the presence of a dysfunctional autonomic arousal system (Banarer and Cryer, 2003). Currently, research suggests that most sleep disturbances related to T2D are largely the result of SDB. OSA is strongly associated with obesity and is likely caused by changes in the upper airway structure in humans (Young et al., 1993). There is a well-defined correlation between OSA and increased risk for T2D due to the presence of decrease glucose tolerance and insulin sensitivity, with a positive correlation between severity of OSA and poorer glucose regulation (Punjabi et al., 2004; Bostros et al., 2009; Aronsohn et al., 2010; Zhang et al., 2018). Similarly, in mice, chronic intermittent hypoxia results in insulin resistance and decreased glucose tolerance (Jiyori et al., 2007). Sleep deprivation, independent of any chronic sleep condition, is also known to disrupt glucose homeostasis. Specifically, sleep restriction causes reductions in glucose tolerance and insulin sensitivity, two components that contribute to increased risk for T2D and are present in early AD (Spiegel et al., 1999). While the mechanisms are not fully understood, there is evidence that sleep fragmentation can lead to altered glucose regulation through decreased cerebral glucose metabolism (Wu et al., 1991), alterations in endocrine function (Van Cauter et al., 2008; Spiegel et al., 2009), and increased inflammatory processes directly tied to T2D risk (Patel et al., 2009). Further, sleep deprivation also increases the expression of ANLS-associated genes, specifically within astrocytes, indicating SWA can regulate neuro-metabolic coupling and any disruption to this process can dysregulate cerebral metabolism independent of pathology (Petit et al., 2013). Selective disruption of slow wave sleep, which is prevalent in both aging and OSA, causes decreased insulin sensitivity and glucose tolerance, suggesting that slow wave sleep plays a specific role in maintaining glucose levels and the risk for T2D (Tasali et al., 2008). Similarly, improving sleep is thought to mitigate some of these metabolic impairments. Administration of orexins, a neuropeptide implicated in regulating both sleep/wake cycles and energy homeostasis, lowered fasting blood glucose levels in streptozotocin(stz)-induced diabetic mice (Tsuneki et al., 2002). In clinical trials, use of a CPAP, the main treatment option for OSA and other sleep breathing disorders, can improve glucose tolerance and lower hemoglobin A_1__C_ levels, a long-term biomarker of blood glucose levels (Babu et al., 2005; Salord et al., 2016).

## Circadian Control of Metabolism and Impact on Sleep

It is worth noting that the circadian system controls the timing of sleep, as mutations in circadian clock genes which alter clock function are associated with sleep phenotypes in mice and humans (Hsu et al., 2015). Fragmentation of normal circadian timing of activity has been observed in preclinical AD and worsens in symptomatic AD in humans (Ju et al., 2013; Musiek et al., 2013; Peter-Derex et al., 2015). Loss of neurons in the suprachiasmatic nucleus (SCN), the master circadian clock of the body, has been described in AD and correlates with loss of circadian rhythms in activity (Swaab et al., 1985; Wang et al., 2015). Thus, degeneration of the circadian system, and the SCN in particular, may be a cause of sleep disruption in AD. Circadian disruption is associated with increased AD risk in humans, and can accelerate Aβ plaque formation in mice (Tranah et al., 2011; Kress et al., 2018). Moreover, the circadian system has strong influences on glucose metabolism, as poor circadian function is a major risk factor for the development of metabolic syndrome and T2D [Bass and Takahashi, 2010; as reviewed in Knutsson and Kempe (2014)]. In mice, a considerable literature links the circadian clock to glucose homeostasis, and mice with clock gene deletions develop obesity and hyperglycemia (Rudic et al., 2004; Turek et al., 2005; Bass and Takahashi, 2010; Marcheva et al., 2010). Thus, circadian rhythm dysfunction is another factor potentially linking sleep, metabolism, and AD.

## Current Therapeutic Options

As previously discussed, treatment of OSA with CPAP to promote increased sleep efficiency can improve glucose tolerance and overall metabolic health (Babu et al., 2005; Salord et al., 2016) as well as AD biomarkers (Ju et al., 2019). Other treatments for T2D have also shown promising effect to alleviating some of the negative impacts on sleep. Metformin, the antihyperglycemic drug and first-line treatment for T2D, reduces hepatic gluconeogenesis, plasma insulin, and insulin resistance (Viollet et al., 2012). Further, individuals treated with metformin show improved sleep quality and quantity, suggesting that maintaining glucose homeostasis is essential for efficient sleep, although the exact mechanisms are not yet understood (Kaibaf et al., 2013). Treatment with insulin therapies has been linked to worse subjective sleep quality, potentially though hyperactivity in the sympathetic nervous system, although this treatment type has been widely understudied in terms of the effects on sleep (Song et al., 2013). Similarly, treating sleep disturbances has been shown to improve aberrant glucose metabolism. Orexins are critical for the regulation of glucose homeostasis and maintenance of the diurnal rhythm via influence over hepatic gluconeogenesis, making the timing modulation of the orexin system crucial. Administration of dual orexin receptor antagonist (DORA) drugs in accordance with the orexin rhythm can improve sleep and glucose tolerance, likely though suppression of hepatic gluconeogenesis rather than an effect on insulin production or sensitivity (Tsuneki et al., 2016). Additionally, results from Merck’s Phase 3 Clinical Trial DORA, suvorexant, improved sleep quality and quantity in individuals with AD. Sleep loss is known to promote the release of Aβ and tau, potentially suggesting that treating these sleep problems could slow disease progression, although no studies of this nature have been done (Herring et al., 2019). While more research on the subject is needed, sleep represents a modifiable aspect of these two diseases and offers a potential therapeutic benefit in delaying the onset of AD and mitigating the increased risk of AD among individuals with T2D by restoring glucose homeostasis.

## Conclusion

**FIGURE 1:**
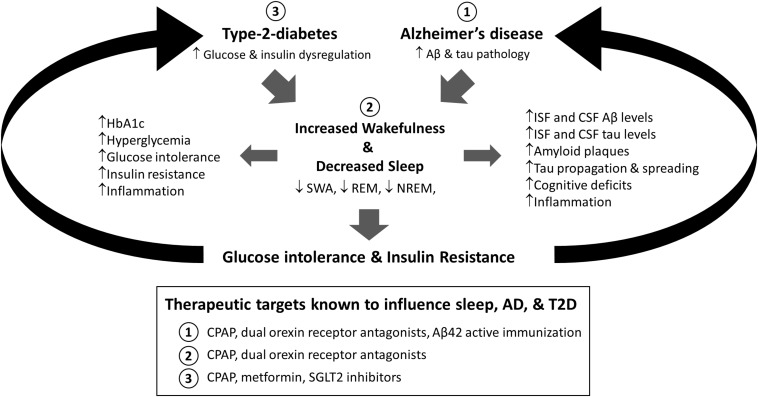
Proposed model for the relationship between sleep, type-2-diabetes (T2D), and Alzheimer’s disease (AD). Both alterations in glucose and insulin homeostasis associated with T2D and Aβ and tau aggregation found in AD are associated with increased sleep disruption. Moreover, sleep deprivation is associated with increased AD-related pathology, including Aβ and tau pathology, cognitive deficits, and inflammation, as well as diabetes related pathology, including metabolic dysregulation, glucose intolerance, and insulin resistance. Therefore, sleep is a modifiable risk factor in T2D and AD that could bidirectionally impact disease progression. Moreover, targeting sleep, AD, or T2D has been shown to modify these relationships (Box: 1, 2, 3). HbA1c = Hemoglobin A1c; SWA = slow wave activity; REM = rapid eye movement sleep; NREM = non-rapid eye movement sleep; Aβ = amyloid-beta; ISF = interstitial fluid; CSF = cerebrospinal fluid; CPAP = continuous positive airway pressure; SGLT2 = sodium glucose cotransporter 2.

This review outlines the interactions between sleep, altered glucose metabolism, and Alzheimer’s disease and suggests the emergence of a feedforward cascade where sleep dysfunction and metabolic impairment contribute to the progression of AD pathology (Figure 1). Similarly, we provide evidence of AD pathology leading to further disruptions to sleep and metabolic health. Therefore, exploring the timing and mechanisms that lead to the emergence of sleep disturbances in both conditions warrants further study as it represents a potential therapeutic treatment option for resolving peripheral glucose intolerance and insulin resistance in individuals with T2D and potentially slowing AD progression.

## Author Contributions

CC and SM conceived of the idea and equally contributed to the writing of the manuscript.

## Conflict of Interest Statement

The authors declare that the research was conducted in the absence of any commercial or financial relationships that could be construed as a potential conflict of interest.
